# Clinical, histopathological and parasitological follow-up of dogs naturally infected by *Leishmania infantum* before and after miltefosine treatment and associated therapies

**DOI:** 10.1371/journal.pone.0313167

**Published:** 2025-01-09

**Authors:** Amábilli de Souza Rosar, Carolina Leite Martins, Álvaro Menin, Carolina Reck, Edmundo Carlos Grisard, Glauber Wagner, Mário Steindel, Patricia Hermes Stoco, Patricia Flavia Quaresma

**Affiliations:** 1 Department of Microbiology, Immunology and Parasitology, Laboratory of Protozoology, Federal University of Santa Catarina, Florianópolis, SC, Brazil; 2 Department of Biosciences and One Health, Federal University of Santa Catarina, Curitibanos, SC, Brazil; 3 VERTÁ, Laboratory of Veterinary Diagnostic, Institute of Veterinary Research and Diagnostic, Curitibanos, SC, Brazil; Tehran University of Medical Sciences, ISLAMIC REPUBLIC OF IRAN

## Abstract

In Brazil, Visceral Leishmaniases is caused by *Leishmania infantum*, and domestic dogs are the main reservoirs in its urban transmission cycle. As an alternative to euthanizing dogs, miltefosine has been used to treat canine visceral leishmaniasis since 2016. In this study, we have assessed the efficacy of miltefosine for treating canine visceral leishmaniasis in a new endemic area through follow-up of naturally infected dogs was evaluated. The clinical, parasitological, and histopathological characteristics of 21 dogs naturally infected with *L*. *infantum* were assessed at three time points: on the day before initiating miltefosine treatment (T0), immediately after treatment completion (T1), and 6 months after treatment completion (T2). Three dogs were treated exclusively with miltefosine, while eighteen received combination therapy with miltefosine with other treatments such as allopurinol, domperidone and immunotherapy. Skin biopsies were obtained from the abdomen to assess inflammatory responses and to quantify parasite loads using qPCR. The parasites were isolated using aspirates acquired from popliteal lymph nodes. Molecular and parasitological analyses confirmed the presence of *L*. *infantum* in all dogs, validating the effectiveness of skin and lymph node samples for diagnosis. The clinical conditions of the infected animals were improved and the skin parasite load decreased after treatment, even when distinct combination therapies were performed. The histopathological assessment revealed a miltefosine-induced reduction in the inflammatory response and a decrease in amastigotes number. Furthermore, a positive correlation was established between the reduction in parasite load and the enhancement of clinical scoring, as well as a reduction in the skin inflammatory response. Our findings suggest that miltefosine-based combination therapies reduce skin parasite load and improve clinical outcomes, while the dogs treated with miltefosine alone showed increased parasitic load and worsened clinical staging at T2. Considering this data belonging to a recent transmission area, treatment strategy suggests effective in controlling canine visceral leishmaniasis.

## Introduction

Visceral leishmaniasis (VL) is an infectious, non-contagious disease caused by *Leishmania* spp. (Kinetoplastea: Trypanosomatidae) and is considered one of the most neglected zoonoses worldwide [1]. Leishmaniases are endemic in large areas of the tropics, subtropics, and the Mediterranean basin. In these areas, a total of 350 million people is at risk, with 12 million cases of infection. Canine leishmaniasis poses a significant threat, particularly in the Mediterranean basin, where an estimated 2.5 million dogs are infected [1]. VL is caused by *Leishmania donovani* in Asia and Africa, whereas *Leishmania infantum* is the causal agent in Brazil, South and Central America, the Mediterranean Basin, the Middle East, and Central Asia [2]. These parasites are transmitted to a variety of susceptible mammalian hosts by the bite of infected female phlebotomine sandflies (Diptera: Psychodidae) [3].

Canine visceral leishmaniasis (CVL) is considered significant and included in the scope of One Health because it presents a serious disease involving both human and veterinary health, which is associated with a complex transmission cycle posing challenges to the implementation of effective control strategies. The pivotal relation of dogs (*Canis familiaris*) to the urban/domestic transmission cycle of this parasite is widely recognized [4]. In addition to lymphoid organ infection, widespread subcutaneous infection is evident in dogs; hence, dogs, characterized by a high parasite load, serve as reservoirs of *Leishmania* and facilitate the transmission to sandfly vectors [5, 6].

Asymptomatic dogs act as silent reservoirs of *Leishmania* for extended periods and are relevant to the dynamics and epidemiology of parasitic transmission [7, 8]. The high prevalence of subclinical and asymptomatic dogs in endemic areas plays an important role in infection, since the *L*. *infantum* isolated from these dogs have the same virulence and infectivity as those isolated from symptomatic dogs [9]. As CVL caused by *L*. *infantum* precedes human infection, the parasite load on dog skin can be used to assess the infection potential of sandfly vectors associated with dogs [7, 10]. These findings led to the adoption of dog euthanasia as a measure to block *L*. *infantum* transmission among animals and humans; however, its effectiveness has been questioned [2, 11].

Owing to the presence of asymptomatic dogs, the variability of clinical manifestations, and the complexity of achieving sensitive and specific diagnoses pose significant challenges to the effective diagnosis of CVL [7]. The prevailing clinical manifestations in dogs infected with *L*. *infantum* encompass skin lesions, lymphadenopathy, splenomegaly, weight loss, muscle atrophy, ocular changes, epistaxis, onychogryphosis, and anemia [12, 13], collectively, these symptoms suggest CVL. The methods used to diagnose CVL are based on three main categories: parasitological (identification of the parasite), serological (detection of anti-*Leishmania* antibodies), and molecular (amplification and analysis of protozoan DNA) [14]. While false-positive diagnosis of CVL can lead to unnecessary euthanasia of non-infected dogs, false-negative results contribute to the maintenance of silent transmission of the parasite. Hence, highly accurate and specific diagnostic tests are extremely important [15].

Several molecular targets for detecting and quantifying *L*. *infantum* infection in dogs, with or without clinical symptoms, involve PCR. PCR-based analysis targeting multicopy DNA regions, such as kinetoplast DNA (kDNA), supports sensitive and qualitative detection of *L*. *infantum* infection. Additionally, qPCR enables the detection and quantification of parasite load in different tissues, allowing the monitoring of infection in dogs during the treatment period, regardless of their clinical condition [16–18].

The use of miltefosine for the treatment of CVL in Brazil was approved in 2016; this control measure is considered the first alternative to the euthanasia of dogs for CVL management. Miltefosine was originally developed as an antitumor drug, however the exact mechanism underlying its antileishmanial effect needs to be further elucidated. In addition to inhibiting the biosynthesis of the glycosylphosphatidylinositol receptor, a key molecule for the intracellular survival of *Leishmania* spp., miltefosine interferes with *Leishmania*-specific phospholipase and protein kinase C biosynthesis. Moreover, it affects the biosynthesis of glycolipids and membrane glycoproteins in the parasite, leading to apoptosis [7]. Additionally, miltefosine may have immunomodulatory properties beyond its antiparasitic effects [7, 19].

To prolong the duration and improve the effectiveness of treatment, combined therapies involving multiple drugs with synergistic or additive properties are used, which enhances the spectrum of activity and therapeutic efficacy [20, 21]. Allopurinol, a hypoxanthine analog, is hydrolyzed by *Leishmania* spp. into an inosine-analog molecule that is incorporated into RNA molecules instead of ATP during transcription, consequently disrupting normal protein synthesis and parasite replication [22, 23]. Owing to this mode of action, allopurinol exhibits leishmaniostatic activity and low toxicity, and hence, it does not provide parasitological clearance of *L*. *infantum* in dogs if used as a single therapeutic drug [22, 24]. Furthermore, domperidone is an antagonist of dopamine receptors acting as an immunomodulator that promotes the release of serotonin and stimulates the production of prolactin [25, 26], increases Th1 lymphocytes, cytokines such as IL-2, IL-12, INF-γ, and TNF-α, leading to macrophage activation and downregulation of the Th2 population [27]. The treatment-induced reduction in parasite load on dog skin may contribute to reduced sandfly infection, and therefore, can suppress the prevalence of leishmaniasis in both dogs and humans residing in endemic areas.

In 2010, autochthonous cases of CVL were registered in the island city of Florianópolis, Santa Catarina State, Brazil [28], followed by the first report of human VL case in 2017 [29]. This city has a unique epidemiological scenario of VL, principally owing to the rapidly growing number of symptomatic dogs and the absence of *Lutzomyia longipalpis*, the most important vector of *L*. *infantum* in Brazil. Other species of sandflies have also been identified as possible vectors of *L*. *infantum*, mainly in areas where *L*. *longipalpis* is not found. Studies identified *Pintomyia fischeri*, *Migonemyia migonei*, and *Nyssomyia neivai* in this region which are considered putative vectors of *L*. *infantum* [30, 31]. Furthermore, a recent study suggests that *Ny*. *neivai* can act as a VL vector transmitting *L*. *infantum* from the state of Santa Catarina [32].

A recent study by Spindola et al. (2024) [33] indicates that the prevalence of CVL in dogs from the Florianópolis region is higher than in other parts of the state. In this study, we investigated the effectiveness of miltefosine, used either solely or in combination, in reducing the parasite load and clinical manifestations in dogs naturally infected with *L*. *infantum* in Florianópolis, Brazil. For this, the parasite load present in the dog’s skin was measured using real-time PCR and a clinical signs scoring system was used to monitor the disease evolution. From this, it is possible to determine whether treatment with miltefosine leads to a considerable reduction in the parasite load and symptoms, which could be evaluated as a possible measure to control VL.

## Methods

### Animals

This study was approved by the Committee on Ethics in Animal Use of the Federal University of Santa Catarina (protocol number: 6251290719). Dogs were enrolled in this study considering the inclusion criteria: i) residence in the region of Florianópolis municipality (Santa Catarina State, southern Brazil); ii) confirmed positive diagnosis of CVL through serological methods (screening with immunochromatography and confirmation with indirect immunofluorescence (RIFI) and enzyme linked immunosorbent assay (ELISA); iii) no previous exposure to miltefosine treatment; and iv) owner’s agreement to treat the animal with miltefosine with or without associations, as indicated by the veterinarian. The exclusion criteria for dogs were as follows: i) skin samples exhibiting no parasite DNA via qPCR analysis before miltefosine-based treatment, and ii) death or change in place of residence during the study. The identification of naturally *L*. *infantum*-infected dogs involved collaboration with veterinarians from private clinics, who performed the primary diagnosis of CVL and carried out treatment.

Among 30 CVL-positive dogs selected in this study, nine were excluded following the exclusion criteria, and a total of 21 dogs of diverse breeds, aged from 5 months to 12 years were analyzed; 57.14%, 28.57%, and 14.29% of them exhibited short, medium, and long hair, respectively. In terms of body size, 66.66%, 14.29%, and 19.05% of included dogs were classified as large, medium, and small, respectively. We included dogs living in peridomicile areas (52.38%), exclusively inside the domicile (28.57%), and semi-domiciled ones (19.05%) having access to both indoor and outdoor areas.

### Therapeutic schemes

Treatment schemes for infected animals were prescribed by and administered under the supervision of their respective attending veterinarians. Miltefosine (Milteforan, Virbac; 2 mg/kg of body weight) was orally administered to all animals every 24 hours for 28 days, as recommended by the manufacturer. However, the specific therapeutic schemes varied for different animals and included the use of allopurinol (10–15 mg/kg of body weight, every 12 hours) and/or domperidone (0.5–1.1 mg/kg of body weight, every 12 hours) prescribed for variable periods. Additionally, after completing miltefosine treatment, 3 out of 21 dogs (14.3%) were vaccinated using LeishTec (CEVA Saúde Animal, Brazil). Immunotherapy was carried out using the LeishTec vaccine (CEVA Saúde Animal, Brazil) in accordance with the protocol proposed by Brasileish [34]. Briefly, three subcutaneous injections of a double dose (2ml) of the vaccine totalling 0.20mg of the A2-His recombinant protein were administered at a 21 days interval.

### Clinical sampling

Clinical sampling was performed at specific time points as follows: T0, the day before the initiation of miltefosine treatment; T1, immediately after the completion of miltefosine treatment (30 days) and T2, six months after treatment completion. As only naturally infected dogs were included in this study, no placebo group was considered. For sample collection, the dogs were placed in the dorsal decubitus position. Subsequently, the intact skin of the left middle abdominal region was shaved, followed by asepsis and local anesthesia using 2% lidocaine. Next, two fragments of healthy skin were collected from the mid-abdominal region of each dog using a 5 mm diameter punch.

Imprints were prepared on glass slides for parasitological examination; subsequently, skin biopsy fragments were stored in ethanol for molecular analysis and fixed in 10% neutral-buffered formalin for histopathological analysis. The aspirate of the popliteal lymph node was aseptically collected using a 13 x 0.45 mm needle (26G x ½”) attached to a 1 ml hypodermic syringe. Part of the collected aspirate was transferred to a tube containing 1 ml of Schneider culture medium (Sigma-Aldrich), pH 7.4, supplemented with 5% inactivated fetal bovine serum (Gibco), 2% human male sterile urine, and 1% antibiotics (streptomycin 100 μg/ml, penicillin 500 U/ml and gentamicin 40 mg/ml), and maintained at 26.5°C for further isolation of the parasite. The remaining material was used for slide smear.

### Clinical evaluation

Dogs were initially weighed and clinically evaluated by a single veterinarian at T0, T1, and T2. Hematological and biochemical tests of animals at T0 (hematocrit, total proteins, albumin, globulins, creatinine, urea, alanine aminotransferase, aspartate aminotransferase, alkaline phosphatase) were carried out. Values ranging from 0 (absence) to 5 (severe) were assigned to each of the 23 clinical signs compatible with CVL. The sum of these values calculated for each dog at T0, T1, or T2 was then used along laboratory tests (hematology and biochemistry) and parasite load quantification to compose the clinical staging as proposed by Brasileish and LeishVet [20, 34], which ranged from “I” (less severe disease) to “V” (more severe disease). Owing to the absence of laboratory testing, dogs at T1 and T2 were staged based on clinical examinations and parasite load.

### Parasitological diagnosis

The Giemsa stained popliteal lymph node aspirate smear and imprints acquired from skin fragments were visualized using an optical microscope (1000x) to analyze the presence of amastigote forms. The presence of *Leishmania* promastigotes in cultures obtained from popliteal lymph node aspirates was analyzed weekly for six weeks; positive samples were further cultured using Schneider’s medium for DNA extraction and cryopreservation with liquid nitrogen.

### Histopathological evaluation of skin

Fixed skin biopsy samples obtained from the mid-abdominal region were dehydrated in graded ethanol solutions, embedded in paraffin, and 4 μm thick sections were prepared using an automatic microtome, followed by staining with hematoxylin and eosin (H&E). For the assessment of inflammatory patterns and cell profiles (neutrophils, histiocytes, lymphocytes, and plasmocytes) in the dermis, epidermis, and adnexal structures, 10 microscopic fields per section were analyzed using optical microscopy. Furthermore, *L*. *infantum* amastigotes present in the tissue were quantified.

### DNA extraction from clinical samples

DNA was extracted from skin fragments using a DNeasy Blood & Tissue Kit (Qiagen) following the instructions provided by the manufacturer. The concentration and quality of the DNA were measured through spectrophotometry using a Picodrop Microliter UV/Vis Spectrophotometer (Picodrop). A negative control was included in each extraction set, which comprised all reagents except for the skin sample. All samples were stored at −20°C until use.

### Molecular identification of *Leishmania* species infecting dogs

Parasites that specifically infect dogs were identified via Restriction Fragment Length Polymorphism (PCR-RFLP) analysis of ribosomal RNA internal transcribed spacer region 1 (ITS1) [35]. For this purpose, a fragment (approximately 350 bp) of the ITS1 region was amplified using 1 μM of primers LITSR 5’ CTG GAT CAT TTT CCG ATG 3’ and L5.8S 5’ TGA TAC CAC TTA TCG CAC TT3’, 50 ng of template DNA, and 1U of GoTaq^.^ DNA polymerase (Promega) in a buffer provided by the manufacturer on a Veriti thermocycler (Applied Biosystems). The following thermal profile for 33 cycles was used: denaturation at 95°C for 30 seconds, annealing of primers at 53°C for 1 min, and extension at 72°C for 1 minute. The amplified products were resolved through electrophoresis using 2% agarose gel, stained with ethidium bromide (10 mg/ml), and digitally analyzed. The amplicons were digested with 1 U *Hae*III (Promega), and the resulting restriction profile was resolved on a 10% polyacrylamide gel and stained with ethidium bromide. DNA samples acquired from *L*. *amazonensis* (IFLA/BR/67/ PH8), *L*. *braziliensis* (MHOM/BR/75/M2903), and *L*. *infantum* (MHOM/BR/74/PP75) reference strains were used as controls in each reaction set; the negative reaction control contained no DNA.

### Quantification of parasite load in the skin of *L*. *infantum*-infected dogs before and after treatment

Parasite load was quantified based on the qPCR protocol following a method reported by Chagas et al. (2021) [6]. For this analysis, we used the following primers 152:5’(C/G)(C/G)(G/C) CC(C/A) CTA T(T/A)T TAC ACC AAC CCC3´and 150:5’GGG GAG GGG CGT TCT GCG AA 3’ that target the conserved region of *Leishmania* kDNA minicircle to amplify a 120 bp fragment [36]. The qPCR standard curve was obtained using 10-fold DNA dilutions up to the equivalent of 10 promastigotes of L. *infantum* MHOM/BR/1974/PP75 reference strain. Each qPCR reaction mix comprised 20 ng of DNA, 0,4 μM of each primer, and 1X of GoTaq qPCR Master Mix (Promega) in a final volume of 10 μl. Amplification was performed on a 7900HT Fast Real-Time PCR System (Applied Biosystems) using the following thermal conditions: denaturation at 95°C for 10 minutes, followed by 35 cycles of 95°C for 15 seconds and 60°C for 1 minute, when fluorescence was acquired. A dissociation curve was generated with an additional cycle, in which temperature increases from 60°C to 95°C at a rate of 0.3°C per second, with continuous fluorescence acquisition. Based on these assays, the cut-off for the detection limit was defined as the 32^nd^ cycle. Every qPCR plate included the standard curve (10^6^–10^1^) as a positive control, and as negative were used the controls from DNA extractions and a blank control (no DNA added); the number of copies was deduced using the standard curve, which was used to calculate the number of parasites per milligram of skin tissue. Using a correction factor, the number of parasites was normalized to the amount of tissue used for DNA extraction and the DNA concentration used for qPCR. The results were expressed as the number of parasites/mg of skin tissue and subsequently transformed into a natural logarithm [37, 38].

### Statistical analysis

The correlation between the clinical status (quantified by clinical staging values) and parasite load before (T0) and after treatment (T1 and T2) was evaluated using Friedman’s nonparametric statistical test. Dunn’s post-hoc test combined with Friedman’s test was used to conduct multiple comparisons and assess the significance at different time points. For all analyses, statistical significance was set at *p*<0.05.

## Results

### Distinct therapeutic schemes and clinical evaluation

Veterinarians have adopted different therapeutic schemes to treat CVL. All professionals overseeing these dogs uniformly prescribed Miltefosine following the manufacturer’s recommendations. In addition to miltefosine monotherapy, combination therapies involving allopurinol, domperidone, immunotherapy, etc. were prescribed (Table 1).

**Table 1:** Miltefosine-based therapeutic schemes adopted for CVL treatment of dogs included in this study.

Groups	Dog No	Miltefosine	Allopurinol/ Duration	Domperidone/ Duration	Immunotherapy	Other associations
			(Days)	(Days)		
G1	1	Yes	Yes/180	Yes/60	No	Omega-3
	2	Yes	Yes/180	Yes/60	No	-
	3	Yes	Yes/180	Yes/60	No	-
	4	Yes	Yes/180	Yes/60	No	Omega-3
	8	Yes	Yes/180	Yes/60	No	-
	10	Yes	Yes/60	Yes/60	No	-
	11	Yes	Yes/180	Yes/90	No	-
	12	Yes	Yes/180	Yes/30	No	-
	13	Yes	Yes/30	Yes/30	No	Omega-3
	14	Yes	Yes/180	Yes/90	No	-
	15	Yes	Yes/60	Yes/30	No	-
	16	Yes	Yes/180	Yes/60	No	-
	18	Yes	Yes/180	Yes/60	No	-
	19	Yes	Yes/180	Yes/60	No	-
G2	5	Yes	No	No	Yes	-
G3	6	Yes	Yes/30	Yes/180	Yes	Marbofloxacin
	17	Yes	Yes/180	Yes/180	Yes	-
G4	7	Yes	Yes/180	No	No	-
G5	9	Yes	No	No	No	-
	20	Yes	No	No	No	-
	21	Yes	No	No	No	-

G1: miltefosine + allopurinol + domperidone; G2: miltefosine + allopurinol; G3: miltefosine + allopurinol + domperidone + immunotherapy; G4: miltefosine + immunotherapy; G5: miltefosine.

At T0, the common clinical signs of CVL were detected in 85% of the dogs, whereas, three animals were asymptomatic. Among clinical signs, hyperkeratosis (95%), lymphadenopathy (90%), onychogryphosis (90%), and dermatitis (85%) were most frequent before treatment (T0) (Table 2). In all symptomatic animals, the clinical signs, especially lymphadenopathy, dermatitis, alopecia, and hyperkeratosis, were reduced at T1 and T2.

**Table 2:** Percentage of clinical signs of CVL observed in 21 dogs.

Clinical signs (%)	T0	T1	T2
Hyperkeratosis	95	80	66
Onychogryphosis	90	90	85
Lymphadenopathy	90	76	76
Dermatitis	85	76	33
Alopecia	76	61	52
Ear tip injury	71	66	52
Hypocolored mucosa	57	42	28
Peeling	47	38	14
Affected mobility	42	42	23
Blepharitis	42	28	28
Keratoconjunctivitis	38	33	9
Hepatomegaly	33	33	38
Splenomegaly	33	33	38
Facial myotrophy	28	28	19
Hyperpigmentation	28	28	19
Swollen paws	28	23	19
Depigmentation	14	14	14
Uveitis	9	9	9
Vomit	4	9	0
Loss of appetite	4	4	0
Corneal opacity	4	0	0
Diarrhea	0	9	0
Epistaxis	0	0	4

The dogs were evaluated before (T0) and after (T1 and T2) treatment with miltefosine and miltefosine plus associations.

**Fig 1:**
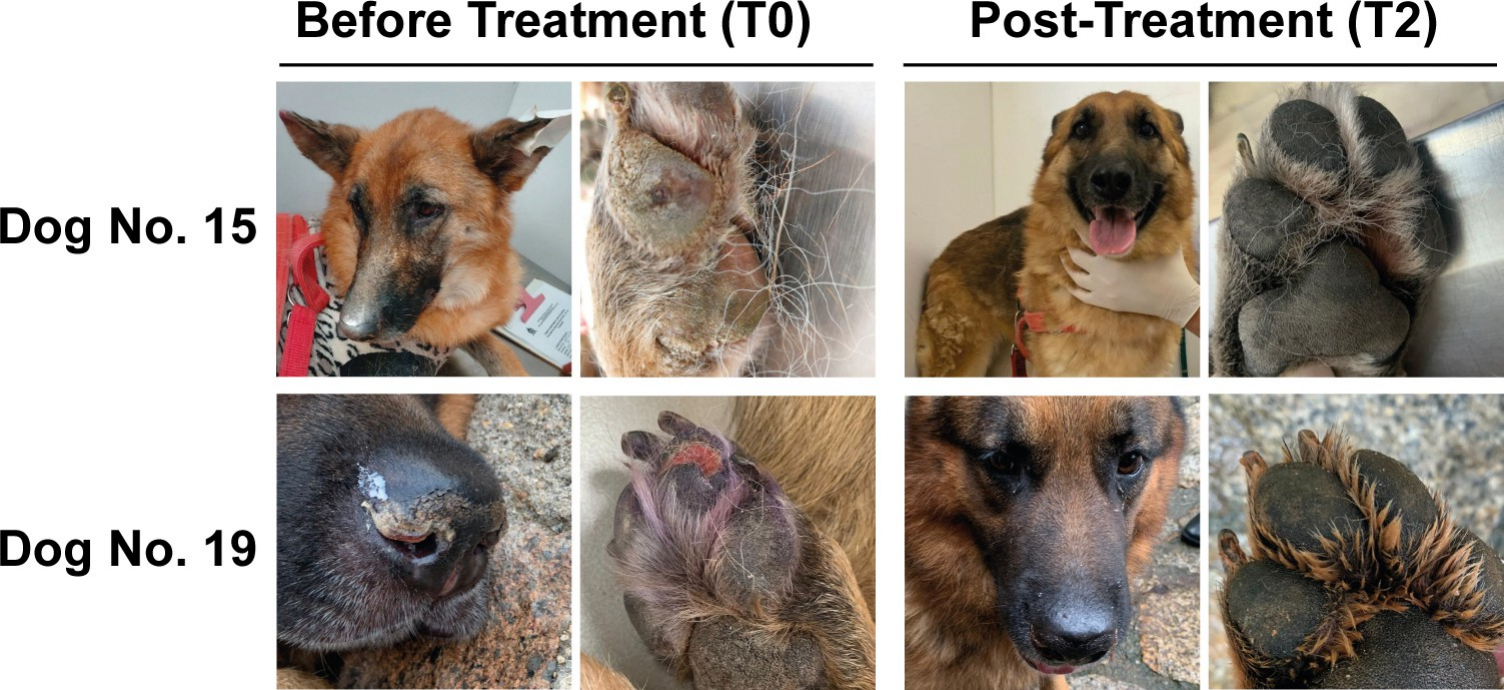
Remission of clinical signs in naturally *L*. *infantum*-infected dogs six months after treatment. This figure illustrates the clinical condition of Dogs 15 and 19, both before and after a six-month treatment period for *L*. *infantum* infection. Dog 15 presented with clinical signs including apathy, weight loss, ear tip injury, and pad hyperkeratosis prior to treatment and dog 19 exhibited nasal hyperkeratosis and pad hyperkeratosis before treatment. The findings indicate that both monotherapy with miltefosine and associated treatments were effective in promoting clinical improvement in dogs infected with *L*. *infantum*.

The clinical aspects of dogs were compared at T0, T1, and T2 using the scoring scale (S1 Table). The overall average of clinical scores before treatment was 19.42 points, which progressively decreased after treatment to 12.85 points in T1 and 8.33 points in T2. Two dogs (No 9 and 21) exclusively treated with miltefosine and one dog (No 2) treated with miltefosine, allopurinol and domperidone exhibited higher score points in T2 compared to T1. Notably, despite the use of various therapeutic schemes, six months after treatment no dog exhibited an increase in their clinical scores compared with before treatment. A representation of the clinical improvements observed six months after treatment (T2) with miltefosine and associations is shown in Fig 1.

**Fig 2:**
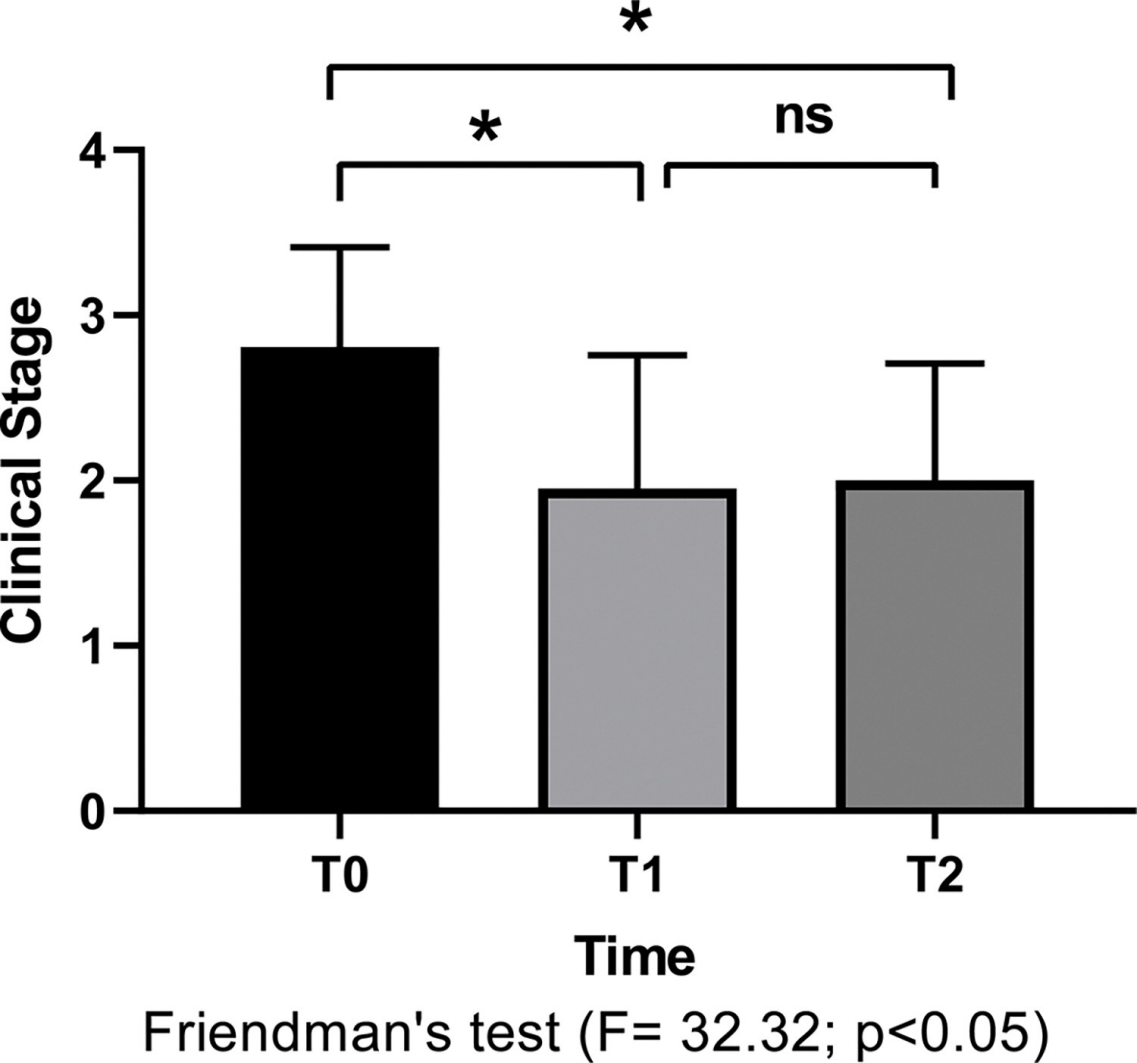
Comparative analysis of clinical stages in naturally dogs infected by *Leishmania infantum* before and after treatment. The clinical stages (stage I to V) were compared before (T0); immediately after miltefosine treatment completion (T1) and six months after the treatment completion (T2). The dogs received miltefosine either as monotherapy or in combinations with other treatments. The analysis employed Friedman’s test along with multiple comparisons (Dunn’s test), utilizing a significance level of p<0.05. Results indicate that treatment with miltefosine, both alone or in combination, led to a significant improvement in the clinical stages of dogs infected with *L*. *infantum*. A marked reduction in clinical symptoms was observed shortly after the completion of treatment (T1) with this improvement being maintained up to six months after treatment (T2).

Along with clinical sign scores, laboratory test results (S2 Table), and parasite load were considered to determine clinical staging. A reduction in the number of dogs at the clinical stages 3–5 and an increase in the number of dogs classified as stage 1 were observed after treatment (Table 3). A comparative analysis of the average clinical stage recorded before and after treatment revealed a reduction in the dogs’ clinical status score after treatment (T1 and T2) months compared to that before treatment (T0) (Fig 2). The clinical stage of dogs evaluated at T1 and T2 did not significantly differ; these results indicate that animals sustained improved clinical status after treatment, regardless of whether monotherapy or miltefosine plus combinations were used. At T0, T1, and T2, 76.2% of the dogs showed weight gain, whereas 23.8% showed a decrease in body weight at T1 and this proportion did not vary (76.2% increase and 23.8% decrease) at T2 compared with that recorded at T0. However, a significant difference in the average body weight was observed only between the dogs before (T0) and six months after treatment (T2) (S1 Fig.).

**Table 3:** Distribution of the 21 dogs naturally infected with *L*. *infantum* based on their clinical staging.

Stage	T0		T1		T2	
	No of dogs	%	No of dogs	%	No of dogs	%
**I**	3	14.2	7	33.3	6	28.6
**II**	5	23.8	12	57.2	13	61.9
**III**	11	52.5	0	0	0	0
**IV**	2	9.5	2	9.5	2	9.5
**V**	0	0	0	0	0	0

The clinical staging before (T0) and after (T1 and T2) treatment according to the Brasileish criteria were as follows: I = no disease, II = no disease/mild disease, III = moderate disease, IV = severe disease, and V = very severe disease.

### Parasitological diagnosis, isolation, and identification of *Leishmania*

PCR-RFLP-based identification of *L*. *infantum* using skin samples revealed that all dogs were positive; which excludes the possibility of co-infection with other sympatric *Leishmania* spp. (S2 Fig.).

The parasitological diagnosis using optical microscopy indicated positivity in 66% [13] of dogs at T0, whereas, among the seven negatively diagnosed dogs, *L*. *infantum* was isolated from five using popliteal lymph node aspirate cultures, and two dogs exhibited negative results (Table 4 and S3 Table). Fifteen (71%), nine (43%), and seven (33%) *L*. *infantum* strains were successfully isolated from lymph node aspirates at T0, T1, and T2, respectively.

**Table 4:** Parasitological and molecular diagnosis of *Leishmania infantum* infected dogs.

	Parasitological		Molecular target	
	Microscopy	Isolation	ITS1	kDNA
Positive	14	15	21	21
Negative	7	6	0	0
Total	21	21	21	21

Diagnosis before miltefosine treatment (T0) was performed using distinct parasitological methods and molecular targets.

### Histopathology of skin biopsies

**Fig 3:**
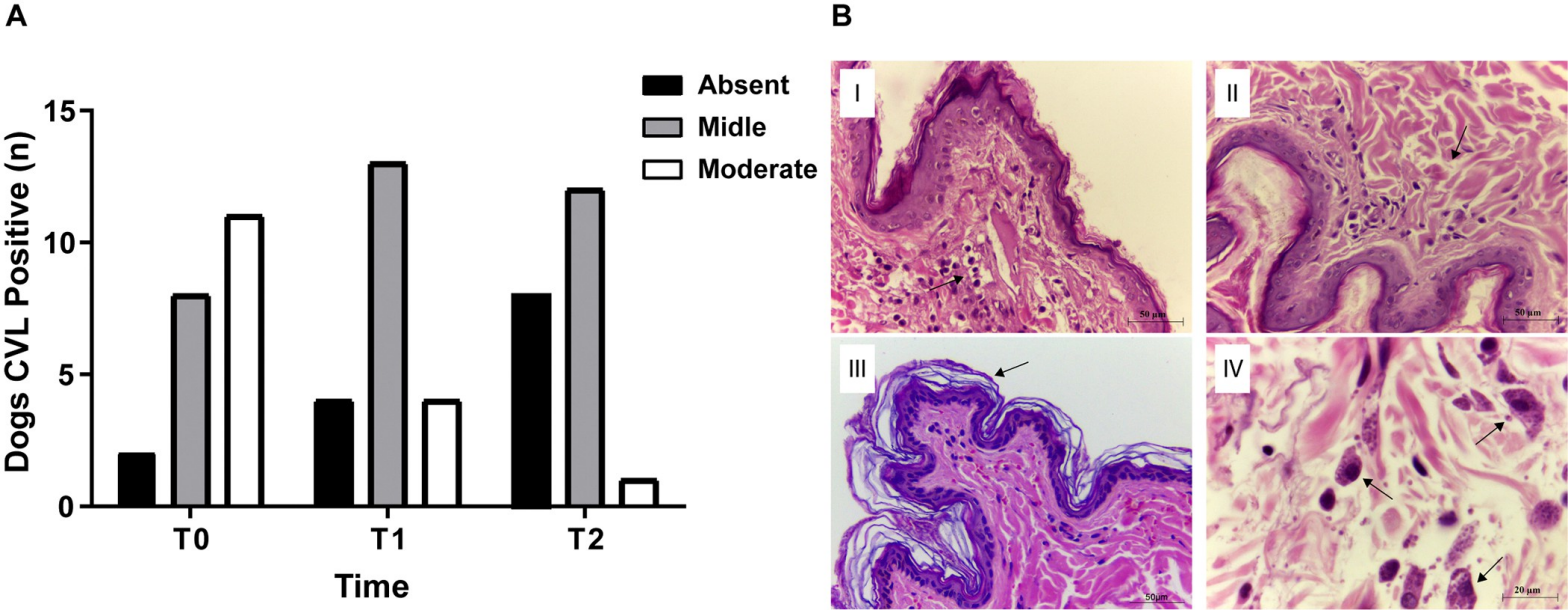
Representative histopathological findings of skin samples from naturally *L*. *infantum*-infected dogs before and after treatment. A) This panel illustrate the Intensity of the inflammatory response in the skin of CVL-positive dogs before (T0) and after treatment (T1 and T2); B) Black arrows highlight key features: (I) inflammatory infiltrate pattern at T0, (II) Inflammatory infiltrate pattern immediately after treatment completion at T1, (III) presence of hyperkeratosis at T0, and (IV) presence of macrophages containing *L*. *infantum* amastigotes in the skin of dogs with CVL at T0.

We investigated the histological pattern of the inflammatory response (intensity and cell profile) through systematic histomorphological analysis using intact skin of *L*. *infantum*-infected dogs before and after treatment (Fig 3A and S4 Table). The inflammatory cell (neutrophils, histiocytes, lymphocytes, and plasmocytes) profile revealed an average of 23.14 leukocytes/field at T0, which further decreased to 19.19 leukocytes/field at T1 and 11.80 leukocytes/field at T2. Notably, a reduction in the intensity of the inflammatory pattern in the skin of a dog with CVL was observed after miltefosine treatment completion (T1) (Fig 3B, 3BI and 3BII).

Furthermore, at T0, hyperkeratosis of the stratum corneum was observed in 11 samples (52.38%) (Fig 3B and 3BIII), whereas, acanthosis (thickening of the spinous layer) was detected in eight samples (38.09%).

*L*. *infantum* amastigotes (Fig 3B and 3BIV) were detected in 38.09% and 19.05% of the samples at T0 and T1, respectively. The average number of *L*. *infantum* amastigotes was 2.9/field at T0, which decreased to 0.76/field at T1. No amastigotes were detected at T2.

### Analysis of parasite load in the skin of dogs with CVL before and after treatment

**Fig 4:**
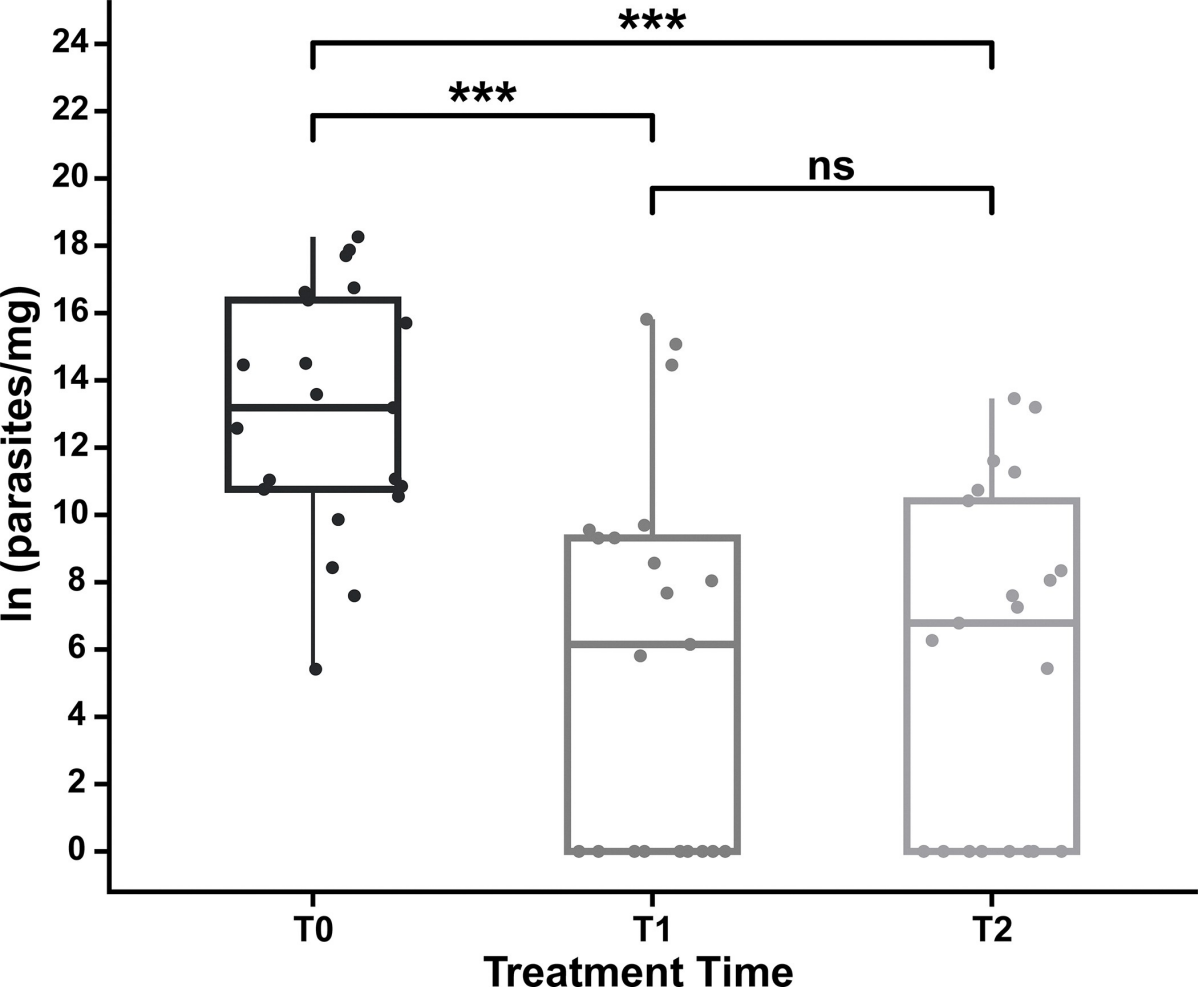
Skin parasite load is reduced in dogs infected by *L*. *infantum* after treatment. Box plot illustrating the average parasite load per milligram of skin CVL-positive dogs before (T0), immediately after treatment (T1) and six months after treatment (T2). The parasite load was measured using quantitative PCR (qPCR), with results indicating a significant reduction in parasite load on the skin of *L*. *infantum*-infected dogs following treatment, as demonstrated by the mean values (***p < 0.001; ns, not significant).

The qPCR-based quantification of *L*. *infantum* revealed that the skin samples were positive in all 21 dogs at T0; the wide range of parasite loads was identified and expressed as the number of parasites/mg of skin tissue (225–85,523,884) (Table 5). In all dogs, parasite load significantly decreased at T1 compared to that at T0, suggesting the efficacy of the treatments (Fig 4); furthermore, parasite load significantly reduced (p< 0.05) in 85.7% of dogs at T2, and no parasites were detected in eight dogs through qPCR. Parasite loads did not significantly differ between T1 and T2, indicating the efficacy of treatment until six months. However, 14.3% of dogs relapsed six months after treatment as revealed an increase in parasite load at T2 (Table 5), corresponding to three dogs exclusively treated with miltefosine.

**Table 5:** Quantification of the parasite load in the skin biopsies of dogs with CVL.

Dog No	Parasitic load (No of parasites/mg skin tissue)			Parasite load -reduction/+increase (%)	
	T0	T1	T2	T1 / T0	T2 / T0
1	533.437	ND	ND	**-** 100.00	**-** 100.00
2	1.991	ND	ND	**-** 100.00	**-** 100.00
3	62.089	3.105	ND	**-** 94.99	**-** 100.00
4	85.523.884	11.053	4.202	**-** 99.98	**-** 99,99
5	19.185	5.257	3.158	**-** 72.59	**-** 83.53
6	791.608	334	1.998	**-** 99.95	**-** 99.74
7	57.821.296	3.521.316	539.733	**-** 93.91	**-** 99.06
8	1.904.211	ND	ND	**-** 100.00	**-** 100.00
9	4.599	ND	33.510	**-** 100.00	**+** 728.63
10	48.928.000	ND	ND	**-** 100.00	**-** 100.00
11	16.504.370	1.896.106	45.994	**-** 88.51	**-** 99.72
12	18.806.153	7.376.765	ND	**-** 60.77	**-** 100.00
13	38.240	ND	527,77	**-** 100.00	**-** 98.62
14	47.164	14.080	ND	**-** 70.14	**-** 100.00
15	1.991.644	469	1.416	**-** 99.97	**-** 99.93
16	51.599.00	ND	ND	**-** 100.00	**-** 100.00
17	6.607.889	11.123	885	**-** 99.83	**-** 99.99
18	289.059	ND	109.675	**-** 100.00	**-** 62.06
19	13.079.390	2.159	78.535	**-** 99.98	**-** 99.40
20	225	ND	229,5	**-** 100.00	**+** 2.00
21	64.267	16.192	700.160	**-** 74.80	**+** 1089.45

Quantification was performed using qPCR before (T0) and after (T1, T2) treatment with miltefosine or miltefosine in associations by qPCR. Absolute values indicate the number of parasites/mg of skin tissue, and the percentage of variation in parasite load at different times after treatment. ND = Not detected.

**Fig 5:**
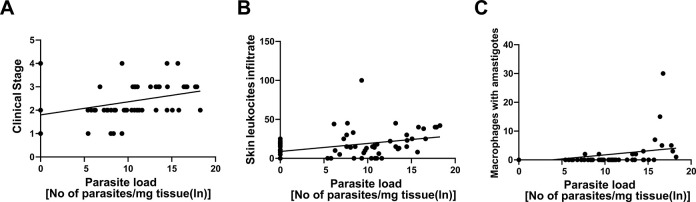
Correlation between skin parasite load and variables in *L*. *infantum*-infected dogs evaluated before and after treatment. The variations in A) clinical staging (*p* = 0.0009), B) mean leukocyte count (*p* = 0.0054), and C) presence of *L*. *infantum*-containing macrophages (*p* = 0.002) were compared.These variables may serve as indicators of the severity of the infection and the efficacy of the treatment administered.

Furthermore, positive correlations of the parasite load with clinical staging (Fig 5A), average leukocyte count (Fig 5B), and the presence of macrophages containing amastigotes were established (Fig 5C).

## Discussion

Currently, the treatment regimens routinely employed by veterinarians in CVL-endemic areas of Brazil involve the use of miltefosine, a unique leishmanicidal drug authorized for treating dogs with VL in Brazil. However, several particularities make therapeutic practices non-standard, since each veterinarian has the autonomy to prescribe complementary medications, diets and specific practical care. In this study, we monitored the animals during the treatment conducted by their clinician under the specific conditions of each tutor. This unique design of our study did not include standardized conditions, such as nutrition, environment, vaccination, deworming, and treatment of other diseases.

To evaluate the effectiveness of miltefosine and associations (leishmaniostatic and/or immunomodulatory drugs), clinical, parasitological, and histopathological analyses of dogs with VL were performed before and after treatment.

In accordance to former studies, the majority of *L*. *infantum*-infected dogs in our study lived in the peridomicile, where exposure to sandfly vectors is facilitated when compared to the intradomicile [39, 40]. This study exclusively included dogs naturally infected with *L*. *infantum*, which received no treatment before; hence, we assessed the impact of initial treatment on various parameters, including clinical status and parasite load monitoring.

A broad spectrum of clinical signs is associated with CVL, which may include an asymptomatic form and severe generalized manifestations, depending on the virulence of the parasite, the immunity of the host animal, and its affected organs [41]. In our study, hyperkeratosis, onychogryphosis, lymphadenopathy, dermatitis, alopecia, ear tip injury, and hypocolored mucosa presented the predominant clinical signs, which is comparable to previous reports [13, 20, 39]. Considerable variability in the manifestation of clinical signs was identified, with some asymptomatic dogs. Even though our study had few asymptomatic dogs, studies show a high prevalence of *L*. *infantum* infection in asymptomatic dogs in endemic areas [9, 42]. This diversity allowed us to include dogs with different responses to the infection. Our results are consistent with previous reports that suggested the primary clinical signs of CVL as a consequence of parasitism intensity and the subsequent deposition of immune complexes in various body tissues [43, 44].

Notably, *L*. *infantum* was successfully isolated from 71% of popliteal lymph node aspirates before treatment (T0). The isolation of the parasite in culture is considered the gold standard for the diagnosis of leishmaniasis. Parasite isolation supports the confirmed diagnosis as well as specific identification and genetic characterization of the pathogen [6, 44]. Lymph node aspiration is a more rapid and minimally invasive technique than bone marrow puncture [45]. Bone marrow and popliteal lymph node samples do not differ in PCR sensitivity or parasite load [6, 46]. However, our study reflects the effectiveness of popliteal lymph node aspirates as an alternative to bone marrow used for parasite isolation, disease monitoring, and infection diagnosis in dogs; the results indicate the high positivity rate and ease of sample collection. Among the seven dogs with a negative parasitological diagnosis from the skin imprint and popliteal lymph node smears, five exhibited successfully isolated parasites in culture. Two dogs with a negative microscopic examination presented a low parasite load at T0 (1,991 and 225 parasites/mg of skin), demonstrating that quantification by qPCR correlated with the real number of parasites in the tissue rather than only the free DNA.

Co-infection with different *Leishmania* species in dogs is considered an important factor in clinical practice. CVL is mainly caused by *L*. *infantum*; however, other species, such as *L*. *braziliensis* and *L*. *amazonensis* can also infect dogs in different regions [35, 47, 48]. In our study, the identification of *L*. *infantum* in all dogs excluded the possibility of coinfection with other sympatric *Leishmania* spp. Co-infections potentially influence disease severity, host immune response, and the efficacy of treatment strategies. Moreover, it can be associated with a more severe clinical course of CVL, leading to a greater probability of spreading *Leishmania* spp. and the development of resistance to conventional treatments [49, 50].

The inflammatory pattern observed in the skin of dogs with visceral leishmaniasis is often associated with a high parasitic load [51, 52]. Our data demonstrated a variation in the inflammatory pattern throughout the treatment. Despite a cellular profile primarily predominated by neutrophils, histiocytes, lymphocytes, and plasmocytes, the number of inflammatory cells decreased over time, supporting clinical improvement in animals. Previously, similar outcomes were reported in dogs with CVL [53, 54]. Epithelial alterations, such as hyperkeratosis and acanthosis, have been reported in CVL in both apparently normal skin and skin with macroscopic lesions [55, 56].

Advances in molecular diagnosis of dogs infected with *L*. *infantum* have allowed wide use of qPCR to monitor parasite load in dogs with VL [57]. The use of kDNA as a target for the diagnosis of *Leishmania* spp. enhances qPCR sensitivity and specificity, facilitating the detection of *Leishmania* spp. in different tissues [6, 45, 58]. In the present study, the positive PCR results for all 21 dogs suggest a wide distribution of parasites in the intact skin, with a parasite load varying from 225 to 85,523.884 parasites/mg of skin tissue.

Dog skin samples collected from the mid-abdominal region were chosen considering the accessibility, minimally invasive nature, reduced risk of secondary infection, and viability in field conditions. Although skin fragments from the ear are commonly used for diagnosis owing to the presence of lesions [59] this area is highly vascularized, hence, the collection of multiple fragments from this part appears more invasive and challenging than using mid-abdominal tissue, particularly in small animals. Lopes et al. (2020) [60] reported that the diagnosis of CVL using skin samples was comparable to that using spleen, bone marrow, and lymph node samples, regardless of the presence of skin lesions. Skin tissue, used in our study, adequately suits the diagnosis and monitoring of *L*. *infantum* infection in dogs with varied clinical presentations, which included asymptomatic dogs as well as those with different degrees of disease severity. Furthermore, we suggest selecting skin and lymph node aspirate samples rather than bone marrow for both parasitological and molecular diagnostic methods, for a less invasive, safer, and more efficient strategy for monitoring *L*. *infantum* infection. Furthermore, our data highlighted a positive correlation between the quantity of parasite DNA/mg of skin and the clinical staging of dogs; similar results were reported by Chagas et al. (2021) [6] and Pereira et al. (2016) [61]. The skin parasite loads detected before and after treatment with miltefosine or combination drugs were positively correlated with the average leukocyte count and the presence of infected macrophages. The treatments reduced the number of parasites on the skin of infected dogs; this finding significantly contributes to understanding the role of treated dogs as reservoirs or sources of infection for sandfly vectors and whether they could still be considered reservoirs. In the current scenario, this knowledge is crucial for a better understanding of VL transmission patterns and the effective treatment of dogs in endemic areas of Brazil. Moreover, we identified a correlation between a negative parasitological diagnosis and a low parasite load in dogs.

The assessment of treatment response reveals that the initial parasite load does not affect treatment outcome; dogs with a substantial initial parasite load showed a significant reduction in parasite load evaluated after treatment. qPCR revealed that associated therapies (miltefosine + allopurinol + domperidone) induced a lower parasite load sustained after six months of treatment than that achieved through other therapeutic regimens using only one or two drugs, which is similar to the results reported by Vaz et al. (2023) [62]. Although 85% of treated dogs showed a reduction in parasite burden, and no parasites were detected by qPCR in 38% of treated dogs after six months of treatment. Although few dogs were treated with miltefosine alone, they were the only ones to show an increase in parasite burden at T2 compared to T0, with two of them showing a worsening in clinical staging at T2 compared to T1. In contrast, dogs receiving combination therapy demonstrated more significant improvements compared to those treated with monotherapy alone. These results agree with a previous report [63], highlighting a higher effectiveness of the combination of miltefosine, allopurinol and domperidone in treating CVL.

Notably, throughout the six-month follow-up period after treatment with miltefosine and therapeutic combinations in dogs naturally infected with *L*. *infantum*, a substantial reduction in the parasite load and the abundance of inflammatory cells in the skin was detected, leading to the alleviation of clinical signs. These results were consistent with those reported by Manna et al. (2015) [57]. The treatment-associated improvement in the histopathological parameters indicates that the use of miltefosine in the tested dogs contributed to the modulation of immune responses in hosts, which potentially reduce the damage associated with exacerbated pro-inflammatory responses observed before treatment. The significance of canine cutaneous tissue in perpetuating the disease through the parasite cycle was reflected because the treated dogs exhibited lower clinical disease scores, a more favorable prognosis, and a diminished incidence of cutaneous parasitism [7, 10, 50, 64, 65]. Considering these aspects, the effective treatment of CVL emerged as a compelling strategy for the intervention and management of the parasite cycle.

## Conclusions

Our study highlights the efficacy of miltefosine as the primary therapeutic agent suppressing the parasite load and the inflammatory responses in the skin of dogs naturally infected with *L*. *infantum* in a recent endemic. The treatment was effective in controlling the disease in 85% of the dogs for up to six months post-treatment. Despite the small number of samples, the combination of miltefosine with allopurinol and domperidone proved to be the most efficient strategy for maintaining a low parasite load in the animals after treatment. These results suggest the effectiveness of miltefosine and therapeutic combinations as a potential tool to control CVL in recent transmission areas in Brazil, and validate the use of skin and lymph node aspirates as appropriate clinical samples for the diagnosis, isolation, and monitoring of parasite load in dogs infected with *L*. *infantum*.

## Supporting information

S1 Table The scoring scale for clinical signs in dogs participating in the study. (XLSX)

S2 Table Laboratory test results before treatment (T0) of the dogs included in the study. (XLSX)

S3 Table Results of different methods used to detect *Leishmania* in dog samples obtained before treatment. (XLSX)

S4 Table Histological evaluation of dog skin samples before and after miltefosine treatment. (XLSX)

S5 Table (XLSX)

S1 Fig Comparative analysis of the average body weight of dogs infected with *Leishmania infantum* before (T0) and after (T1, T2) treatment with miltefosine or miltefosine-containing combination drugs. (TIF)

S2 Fig Representative *Hae*III digestion profile of the ITS1 marker amplified using DNA obtained from the skin of infected dogs with VL. MW: molecular weight marker (pUC18 digested with *Hae*III); *La*: *L*. *amazonensis* strain (IFLA/BR/67/PH8); *Lb*: *L*. *braziliensis* strain (MHOM/BR/75/M2903); *Li*: *L*. *infantum* strain (MHOM/BR/74/PP75); DNA from skin biopsies of dogs 4, 7, 9, 10, 12, 14, 15, 17, 19, and 20 at T0.
(TIF)

S1 Raw image Representative *Hae*III digestion profile of the ITS1 marker amplified using DNA obtained from the skin of infected dogs with VL. MW: molecular weight marker (pUC18 digested with *Hae*III).
(TIF)
