# Supplementary figures and images for: Clinical, histopathological and parasitological follow-up of dogs naturally infected by Leishmania infantum before and after miltefosine treatment and associated therapies
Source: PLoS One. 2025 Jan 9;20(1):e0313167. doi: 10.1371/journal.pone.0313167 (PMC11717219; doi:10.1371/journal.pone.0313167)

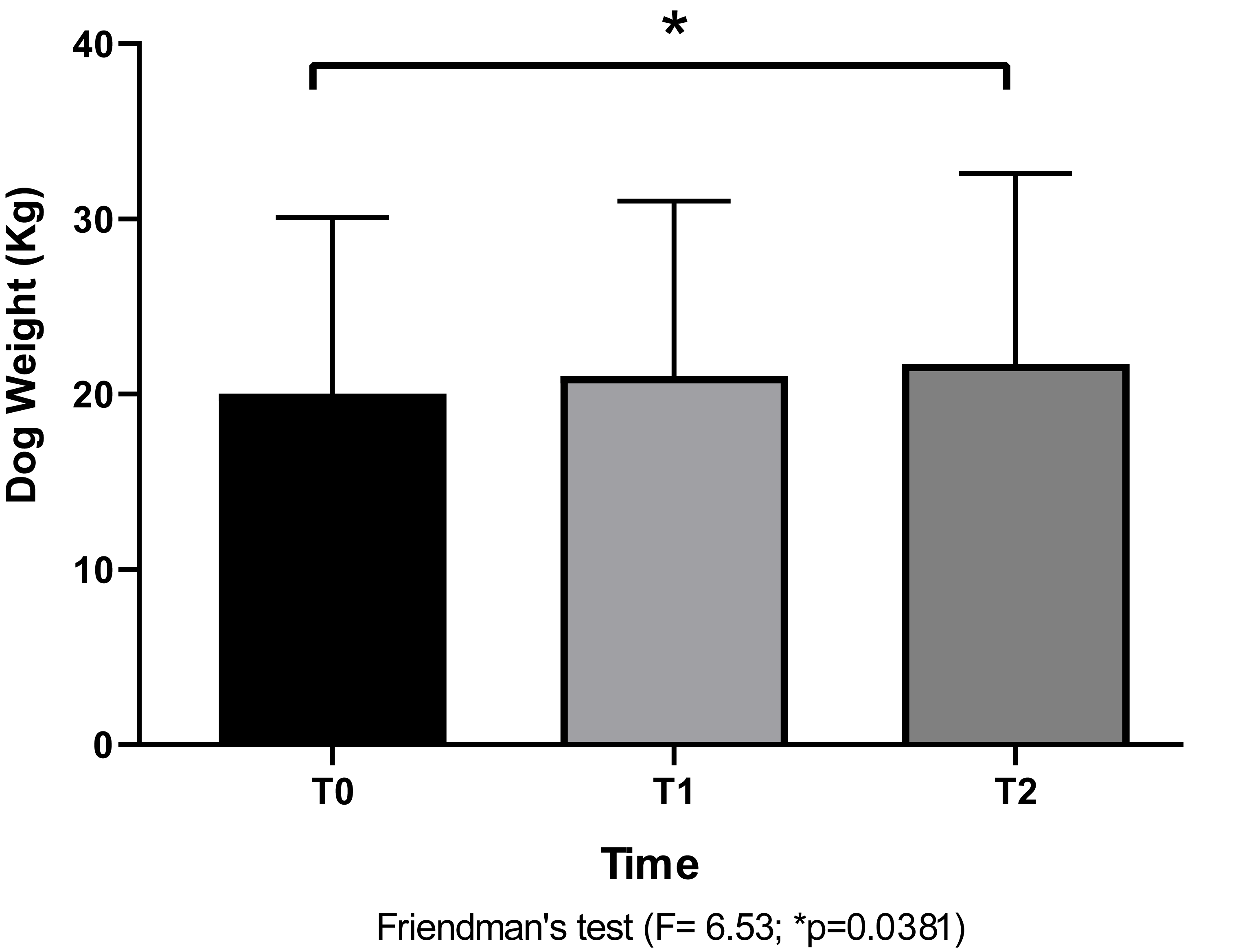

Supplement: S1 Fig — (TIF) [file pone.0313167.s006.tif]

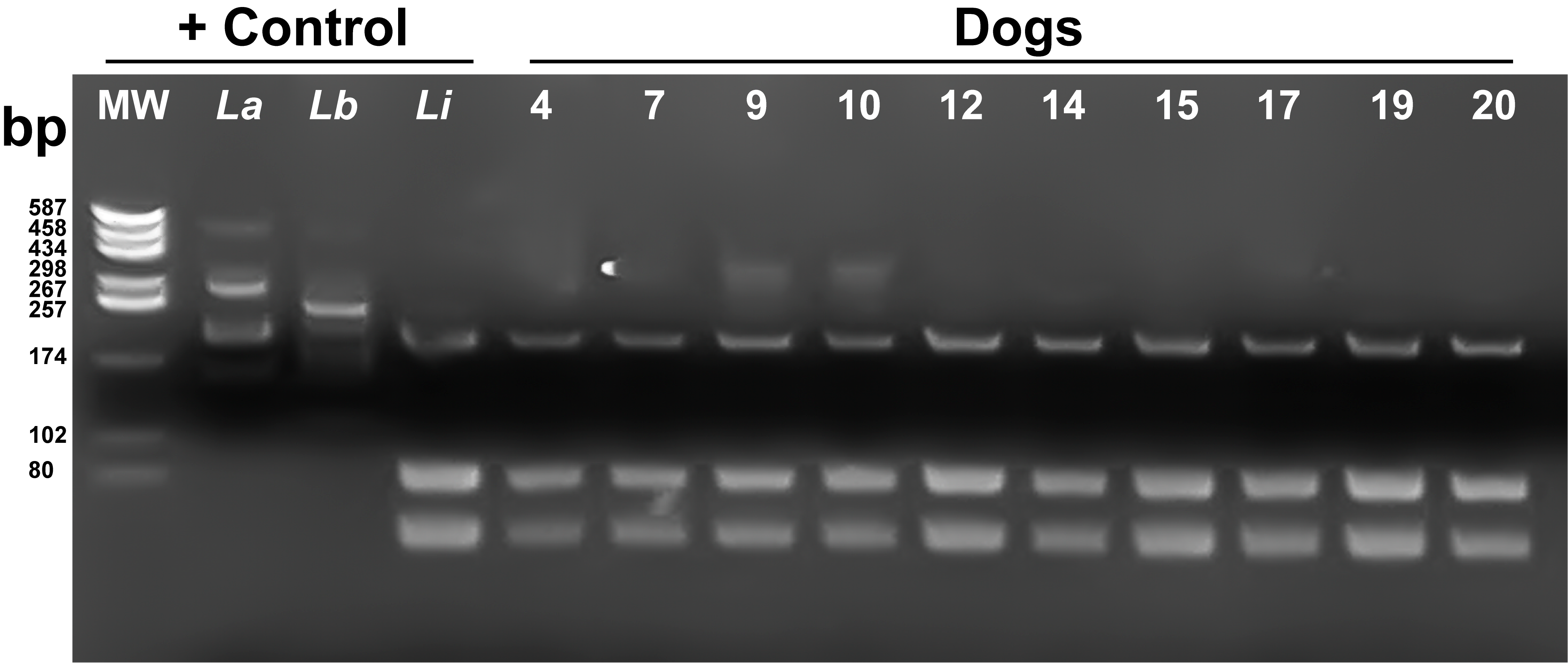

Supplement: S2 Fig — MW: molecular weight marker (pUC18 digested with HaeIII); La: L. amazonensis strain (IFLA/BR/67/PH8); Lb: L. braziliensis strain (MHOM/BR/75/M2903); Li: L. infantum strain (MHOM/BR/74/PP75); DNA from skin biopsies of dogs 4, 7, 9, 10, 12, 14, 15, 17, 19, and 20 at T0. (TIF) [file pone.0313167.s007.tif]
